# The local variation of the Gaussian modulus enables different pathways for fluid lipid vesicle fusion

**DOI:** 10.1038/s41598-023-50922-7

**Published:** 2024-01-02

**Authors:** Matteo Bottacchiari, Mirko Gallo, Marco Bussoletti, Carlo Massimo Casciola

**Affiliations:** Department of Mechanical and Aerospace Engineering, Sapienza Università di Roma, Rome, Italy

**Keywords:** Biological physics, Surfaces, interfaces and thin films, Nonlinear phenomena

## Abstract

Viral infections, fertilization, neurotransmission, and many other fundamental biological processes rely on membrane fusion. Straightforward calculations based on the celebrated Canham–Helfrich elastic model predict a large topological energy barrier that prevents the fusion process from being thermally activated. While such high energy is in accordance with the physical barrier function of lipid membranes, it is difficult to reconcile with the biological mechanisms involved in fusion processes. In this work, we use a Ginzburg–Landau type of free energy that recovers the Canham–Helfrich model in the limit of small width-to-vesicle-extension ratio, with the additional ability to handle topological transitions. We show that a local modification of the Gaussian modulus in the merging region both dramatically lowers the elastic energy barrier and substantially changes the minimal energy pathway for fusion, in accordance with experimental evidence. Therefore, we discuss biological examples in which such a modification might play a crucial role.

## Introduction

Fusion of fluid lipid bilayer membranes is a pivotal process in cell life, involved in fertilization^1^, neurotransmission^2^ or intercellular communications^3^. Enveloped viruses also exploit this process to release their genetic material into cells to be infected^4^. Regardless of the specific biological context, all these processes share general characteristics in membrane fusion, which are also of strong interest in applications, e.g. for the development of antivirals^5^ and drug delivery^6,7^.

The celebrated Canham–Helfrich elastic energy^8,9^,

1 $$\begin{aligned} E_{\textrm{CH}}[\Gamma ] = 2k \int _{\Gamma } (M-m)^2 \; dS + k_{\textrm{G}} \int _{\Gamma } G \; dS, \end{aligned}$$

successfully describes many features of a fluid lipid membrane, representing it as a two-dimensional surface $\Gamma$ corresponding to the bilayer midplane. The energy density depends on the principal curvatures of $\Gamma$, through the mean curvature *M*, and the Gaussian curvature *G*. *m* is the spontaneous mean curvature that the membrane tends to assume in absence of external forces, while *k* and $k_{\textrm{G}}$ are usually two constants, known as bending rigidity and Gaussian modulus, respectively. Therefore, the first contribution to the right-hand side is referred to as the bending energy, while the second one as the Gaussian energy. The energy density can also be rearranged in terms of the isotropic and deviatoric components^10^. It is worth stressing that the Gaussian term has the feature to be a topological invariant, thus remaining constant in absence of fusion (or fission) events. Indeed, the Gauss-Bonnet theorem of differential geometry states that

2 $$\begin{aligned} \int _{\Gamma } G \; dS = 2 \mathrm {\pi } \chi (\Gamma ) - \int _{\partial \Gamma } k_{\textrm{g}} \; dl, \end{aligned}$$

where $\chi (\Gamma )$ is the Euler characteristic of the surface $\Gamma$ and $k_{\textrm{g}}$ is the geodesic curvature of the boundary $\partial \Gamma$. Considering compact surfaces like lipid vesicles, the line integral vanishes since there is no boundary, and $\chi (\Gamma ) = 2(1 - g)$, where *g* is the genus of $\Gamma$, which intuitively counts the holes in the surface, i.e. $g = 0$ for a sphere, $g = 1$ for a torus, and so on. Such a special characteristic leads to a quantized Gaussian energy variation when calculated between an instant before and an instant after a fusion event. Since $-\, k_{\textrm{G}} \approx k \approx 20 \, k_{B}T$^11–14^, where $k_{B}$ is the Boltzmann constant and *T* the temperature, the aforementioned Gaussian energy variation plays a predominant role in a fusion event. Indeed, for example, two spherical vesicles merge through the formation of a catenoid-like neck connecting them^15^. Hence, since a catenoid has zero mean curvature, the variation of the bending energy is negligible when $m = 0$, whereas the Gaussian energy jumps of $-\, 4 \pi k_{\textrm{G}} \approx 250 \, k_{B}T$. This energy cannot be supplied by thermal fluctuations, a fact in accordance with the stability and barrier function of lipid membranes. As a consequence, the fusion process requires the action of external agents, typically proteins, even though $250 \, k_{B} T$ seem to be a too demanding request, see also Deserno^16^. From a Canham-Helfrich theory perspective, a possible solution to lower the elastic energy barrier is to locally modify the Gaussian modulus in the merging region, shifting it towards higher values, while retaining $- \,20 \, k_{B}T$ in the remainder of the vesicles. Indeed, in this case, $k_{\textrm{G}}$ would go under the integral sign, loosening the Gauss-Bonnet theorem constraint, and thus reducing the energy barrier. It is worth noticing that this picture is consistent both with the observation that inducing a negative spontaneous curvature $m^{\textrm{ml}}$ of the constituent monolayers has a fusogenic effect^17^ and the possibility that external agents can modify the monolayers’ bending and Gaussian elastic constants^18^, $k^{\textrm{ml}}$ and $k_{\textrm{G}}^{\textrm{ml}}$, respectively, with the latter being usually negative^19^. Indeed, considering for simplicity two symmetric leaflets, the consistency relation between the elastic energy of a bilayer and of its constituent monolayers reads

3 $$\begin{aligned} k_{\textrm{G}} = 2 \left( k_{\textrm{G}}^{\textrm{ml}} - k^{\textrm{ml}} z_0 m^{\textrm{ml}}\right) , \end{aligned}$$

where $z_0$ is a measure of the bilayer thickness^20^. However, despite its unassailable merits, the Canham–Helfrich model is not able to describe fusion processes, unless cuts are artificially introduced in the surfaces^21^. This makes it impractical to investigate the locally variable $k_{\textrm{G}}$ scenario.

Recently, we have introduced a Ginzburg–Landau type of free energy^22^, which treats the bilayer as a diffuse interface. The Ginzburg-Landau free energy approaches the Canham-Helfrich one in the limit of small width-to-vesicle-extension ratio (sharp-interface limit), and has the additional ability to handle topological transitions, thus allowing access to the merging process and to the computation of the involved forces, even accounting for the otherwise inaccessible component related to the Gaussian energy term^23^. Thence, in the same work, we showed that the minimal free energy pathway (MEP) for the fusion of two large unilamellar vesicles (LUVs) with uniform $k_{\textrm{G}} = -\, k = -\, 20 \, k_{B} T$ has an energy barrier of about $226 \, k_{B} T$, associated with a very steep energy path. Furthermore, we showed that the involved forces are localized in the merging region, corroborating the possibility that a local modification of $k_{\textrm{G}}$ in that region may significantly affect the fusion barrier.

In this work, we investigate the scenario of a locally varying Gaussian modulus, increasing it in the merging region to a value close to the stability limit with respect to saddle deformations of the membrane (or bicontinuous phases)^20^. We compute the MEP for the fusion between two spherical LUVs, a configuration of interest for experiments^24–26^. We show that this local modification not only reduces the overall energy barrier, but also modifies the part of the energy landscape in which fusion intermediates appear, providing results compatible with experimental observations.

## Results

### Free energy with a locally variable Gaussian modulus

As anticipated, in order to locally modify the Gaussian modulus and gain access to the merging process, we use a Ginzburg–Landau type of free energy we have recently introduced^22^, whose main features are briefly recalled here. The free energy exploits a phase-field $\phi (\textbf{x})$, a smooth function defined on a domain $\Omega$. The field assumes its limiting values $\pm \, 1$ in the inner and the outer environment of the vesicles taken into account, while the $\phi (\textbf{x}) = 0$ level set identifies the Canham–Helfrich elastic surface $\Gamma$, that is the membrane mid-surface. The transition between the two limiting values $\pm \, 1$ occurs in a narrow region, the so-called *diffuse interface*, which represents the membrane bilayer, usually about $\ell _{\textrm{me}} = 5 \, \text {nm}$ thick. The diffuse interface width is controlled by a small parameter $\epsilon$, chosen to be such that the bilayer thickness $\ell _{\textrm{me}}$ is $6 \epsilon$. This requirement sets the size of our system and is paramount since the scale invariance to which vesicles are usually subjected is broken during topological transitions. The free energy $E[\phi , \eta ]$ is an integral-type functional depending upon the phase-field $\phi (\textbf{x})$, with the integration done over the entire domain $\Omega$. Here, $\eta (\textbf{x})$ is another, auxiliary field, introduced for the purpose of distinguishing the patch with modified Gaussian modulus from the remainder of the vesicle^27^. Strictly speaking, $E[\phi , \eta ] = E_{\textrm{B}}[\phi ] + E_{\textrm{G}}[\phi , \eta ]$, where

4 $$\begin{aligned} E_{\textrm{B}}[\phi ] = k \, \dfrac{3}{4 \sqrt{2}} \, \epsilon \, \int _{\Omega } \Psi _{\textrm{B}}^2(\textbf{x}) \; dV, \end{aligned}$$

5 $$\begin{aligned} \Psi _{\textrm{B}} = \nabla ^2 \phi - \dfrac{1}{\epsilon ^2} \left( \phi ^2 - 1\right) \left( \phi + \sqrt{2}\epsilon m\right) \;, \end{aligned}$$

and

6 $$\begin{aligned} E_{\textrm{G}}[\phi , \eta ] = \dfrac{35}{16 \sqrt{2}} \, \epsilon ^3 \, \int _{\Omega } k_{\textrm{G}}(\eta (\textbf{x})) \, \Psi _{\textrm{G}}(\textbf{x}) \; dV, \end{aligned}$$

7 $$\begin{aligned} \Psi _{\textrm{G}} = \dfrac{ \varvec{\nabla } |\varvec{\nabla } \phi |^2 \cdot \varvec{\nabla } |\varvec{\nabla } \phi |^2}{2} - \left( \varvec{\nabla } |\varvec{\nabla } \phi |^2 \cdot \varvec{\nabla } \phi \right) \nabla ^2 \phi + |\varvec{\nabla } \phi |^2 \bigg [\left( \nabla ^2 \phi \right) ^2 + \varvec{\nabla } \phi \cdot \varvec{\nabla } \nabla ^2 \phi - \dfrac{\nabla ^2 |\varvec{\nabla } \phi |^2}{2}\bigg ] \;. \end{aligned}$$

$E_{\textrm{B}}[\phi ]$ approaches the bending energy of the membrane in the sharp-interface limit^28–30^, while $E_{\textrm{G}}[\phi , \eta ]$ approaches the Gaussian term^22^. Therefore, $E[\phi , \eta ]$ recovers the Canham-Helfrich free energy in the limit of small width-to-vesicle-extension ratio. In other words, if *A* is the surface area of the vesicle and $D_{\textrm{ve}} = \sqrt{A/\mathrm {\pi }}$ is its characteristic length, then $E[\phi , \eta ] \sim E_{\textrm{CH}}[\Gamma ]$ when $\lambda = \epsilon / D_{\textrm{ve}}<< 1$. One may notice that, in Eq. (6), the Gaussian modulus $k_{\textrm{G}}$ depends on $\textbf{x}$, thus allowing to have $k_{\textrm{G}} = - k = - \,20 \, k_{B} T$ on the whole membrane, except in the merging region where we set $k_{\textrm{G}} \approx 0$, a value close to the bilayer stability limit with respect to saddle deformations^20^. This spatial dependence is introduced through the auxiliary field $\eta (\textbf{x})$, which is needed to follow the motion of the membrane patch with modified Gaussian modulus. Additional details on the auxiliary field, the free energy functional and the adopted numerical scheme are reported in Section Methods.

### Fusion pathway

**Figure 1:**
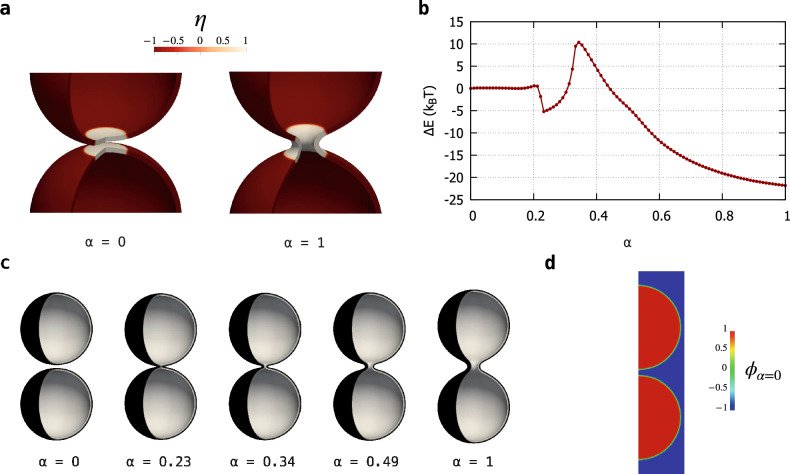
The MEP obtained with the string method for the fusion of two spheres into a prolate shape, $D_{\textrm{ve}} \approx 206 \; \text {nm}$. The path consists of vesicles with $m = 0$, constant total area and enclosed volume, and therefore with constant reduced volume $v = 1/\sqrt{2}$. Simulation assumes z-axial symmetry. (**a**) A zoom of the initial and final configurations, $\phi _{\alpha =0}({\textbf{x}})$ and $\phi _{\alpha =1}({\textbf{x}})$, respectively. The color map shows the values taken by the auxiliary field $\eta (\textbf{x})$ on the membrane. $\eta = +\,1$ identifies the membrane patch with $k_{\textrm{G}} \approx 0$, while $\eta = -\,1$ is the membrane part with $k_{\textrm{G}} = -\, k = - \,20 \, k_{B} T$. The size of the patch with $k_{\textrm{G}} \approx 0$ is conserved along the MEP. (**b**) The free energy difference $\Delta E$ with respect to the initial state along the MEP, namely in function of the normalized arc-length $\alpha$ discretized by means of 100 equidistant images represented by the points, $\alpha = i/99$ with $i = 0, \,..., \, 99$, see also Section Methods. There are three minima, $\alpha = 0, \, 0.23, \, 1$, and a relevant maximum (saddle-point) at $\alpha = 0.34$. (**c**) Vesicle shapes along the pathway as identified by the diffuse interface, namely the transition layer between $\phi = \pm \, 1$. The three minima of the MEP correspond to two disjointed vesicles, a configuration reminiscent of the hemifusion state, and a final prolate shape, respectively. (**d**) The phase-field function in the $r-z$ plane for the initial configuration, namely $\phi _{\alpha =0}({\textbf{x}})$. Here, it is possible to see the small transition layer between $\phi = \pm \, 1$ which identifies the diffuse interface of the two initial disjointed vesicles. An enlargement of the merging region is reported in Fig. 2.

An MEP is a curve on the energy landscape $E[\phi , \eta ]$ connecting two stable vesicle states, which identifies a sequence of vesicle configurations $\phi _\alpha ({\textbf{x}})$ as the normalized arc-length $\alpha \in [0, 1]$ varies. By definition, the curve is such that it is everywhere tangent to the gradient of the potential, except at critical points^31^, namely $\partial \phi _\alpha /\partial \alpha \propto \delta E[\phi _\alpha , \eta _\alpha ]/\delta \phi$ and $\partial \eta _\alpha /\partial \alpha \propto \delta E[\phi _\alpha , \eta _\alpha ]/\delta \eta$, where $\delta E[\phi _\alpha , \eta _\alpha ]/\delta \phi$ and $\delta E[\phi _\alpha , \eta _\alpha ]/\delta \eta$ denote the functional derivatives of $E[\phi , \eta ]$ computed at position $\alpha$ along the curve. Here, the initial and final stable states are two separate spherical vesicles and a prolate one^32^, $\phi _{\alpha =0}(\textbf{x})$ and $\phi _{\alpha =1}(\textbf{x})$, respectively. By means of a rare event technique, the zero temperature string method^33,34^, we compute the MEP by evolving an initial guess for the path discretized in 100 images, referred to as the string. Along the MEP, all the configurations have the same total surface area *A* and enclosed volume *V*, therefore they have the same reduced volume $v = V/(\mathrm {\pi } \, D_\text {ve}^3 / 6) = 1 / \sqrt{2}$, which is the only reduced volume geometrically compatible with the presence of two separate identical spheres^35^, see also Section Methods. Here, the two initial disjointed spherical LUVs have the same diameter of about $146 \, \text {nm}$, therefore $D_\text {ve} \approx 206 \, \text {nm}$ along the MEP. Vesicles are supposed to have zero spontaneous curvature, $m = 0$, while, as anticipated, $k_{\textrm{G}} = -\, k = -\, 20 \, k_{B} T$ except in the merging region where $k_{\textrm{G}} \approx 0$. The initial and final configurations, $\phi _{\alpha =0}({\textbf{x}})$ and $\phi _{\alpha =1}({\textbf{x}})$, respectively, are depicted in Fig. 1a, where the color map shows the values assumed by the auxiliary field. $\eta = +\,1$ identifies the membrane patch with modified Gaussian modulus, $k_{\textrm{G}} \approx 0$, while $\eta = -\,1$ is the membrane part with $k_{\textrm{G}} = -\, k$. As apparent, the transition between the two zones is rapid but smooth, see also Section Methods. The size of the modified patch is conserved along the pathway, Section Methods, and amounts to about $2 \%$ of the total surface area.

Figure 1b shows the computed MEP, providing the Ginzburg-Landau free energy difference $\Delta E = E[\phi _\alpha ] - E[\phi _{\alpha =0}]$ of the membrane along the path, namely as a function of the string parameter $\alpha$. The pathway greatly differs from the one obtained with constant $k_{\textrm{G}}$ in our previous work^22^, where there was only one, steep, and large ($226 \, k_{B} T$) elastic energy barrier that prevented the fusion process (therefore no stable intermediates were present). As apparent, here, the local modification of the Gaussian modulus enables a different pathway, consisting of three stable states (minima of the curve) at $\alpha = 0, \, 0.23, \, 1$ and two saddle-points of the energy landscape (maxima of the curve) at $\alpha = 0.2, \, 0.34$. The first maximum is very small, and separates a sequence of neutral equilibrium states from a downhill stretch that leads to the minimum at $\alpha = 0.23$. In what follows, we will focus on the second saddle-point and neglect the first one, as it does not substantially contribute to the elastic picture that will emerge. The difference between the energy at the saddle-point $\alpha = 0.34$ and the energy at the corresponding preceding minimum, $\alpha = 0.23$, provides the elastic energy barrier that must be overcome in order to complete the fusion process. This energy is about $16 \, k_{B} T$, and increases to $39 \, k_{B} T$ if one considers a bending rigidity *k* of $50 \, k_{B} T$^36^. Indeed, for clarity, Fig. 1b is plotted considering $k = 20 \, k_{B} T$, but energies can be directly rescaled for other bending rigidities, see also Section Methods. These values show that the local modification of $k_{\textrm{G}}$ is able to drastically reduce the work needed to drive the fusion process, lowering the elastic energy barrier to values less than $40 \, k_{B} T$, thus allowing the topological transition to be thermally activated^37,38^. Figure 1c shows some configurations along the MEP: the two vesicles are initially separated ($\alpha = 0$), then brought into close apposition to reach a stable state ($\alpha = 0.23$) which is reminiscent of the experimentally observed hemifusion stalk configuration^25,39^. Subsequently, a catenoid-like neck starts to appear ($\alpha = 0.49$) leading to the formation of a stable pore ($\alpha = 1$). Also catenoid-like necks are experimentally observed^15^. The aforementioned sequence of neutral equilibrium states corresponds to rigid translations during which the two vesicles approach each other before falling in the energy minimum $\alpha = 0.23$ with the hemifusion configuration. This stretch of the MEP correspond to the zeroth stage of fusion as discussed by Smirnova and Müller^40^ and actually involves a dehydration energy barrier which is not considered in this work. Therefore, we have computed an intrinsic elastic MEP, whose barrier can be overcome thermally, while, as we will discuss later, the energy needed for the zeroth stage can be supplied by external agents. As an example, Fig. 1d shows the phase-field function $\phi _{\alpha =0}({\textbf{x}})$ in the $r-z$ plane, whose transition layer between $\phi = \pm \, 1$ identifies the diffuse interface of the initial configuration consisting of two disjointed spheres with z-axial symmetry.

**Figure 2:**
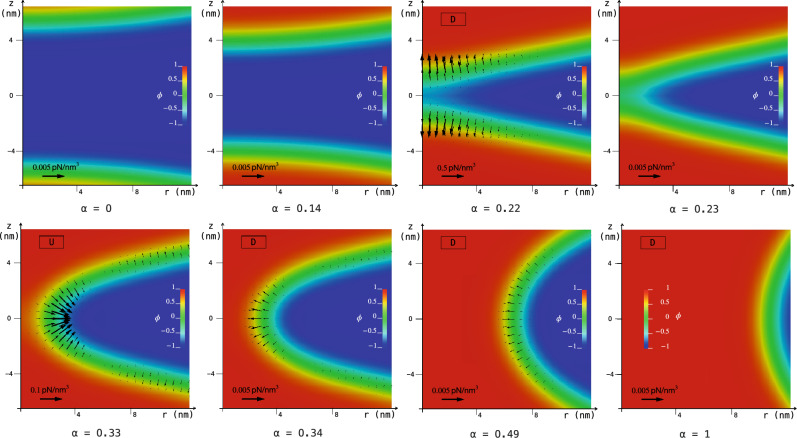
Enlargements of the merging region in the $r-z$ plane. The contour plots show the phase-field $\phi$ in the configuration identified by the normalized arc-length $\alpha$ along the MEP of Fig. 1b. The two vesicles are initially brought into close apposition, then the formation of two bulges ($\alpha = 0.22$) allow to reach a stable hemifusion state ($\alpha = 0.23$). Subsequently, a catenoid-like neck starts to appear ($\alpha = 0.33, \, 0.34, \, 0.49$) leading to the formation of a small stable pore ($\alpha = 1$). Vectors, scaled according to the reference arrow in each plot, depict the force fields $\textbf{f}$ needed to counterbalance the elastic reaction of the membrane in the given configuration. The U- and D-symbols point out the configurations taken along the uphill and downhill stretches of the MEP of Fig. 1b, respectively. The membrane part shown in these enlargements belongs the patch with modified Gaussian modulus, $k_{\textrm{G}} \approx 0$.

Figure 2 shows the enlargement of the merging region along the MEP of Fig. 1. Therefore, the membrane part shown in these enlargements belongs to the patch with modified Gaussian modulus, $k_{\textrm{G}} \approx 0$. The contour plots show the phase-field $\phi (\textbf{x})$ in the $r-z$ plane, and the different configurations along the MEP are identified by $\alpha$. The enlargements allow to identify the presence of two bulges ($\alpha = 0.22$) preceding the hemifusion state ($\alpha = 0.23$), as also observed in experiments^25,41^. Furthermore, the experimentally observed^15^ hourglass-shaped neck is apparent ($\alpha = 0.33, \, 0.34, \, 0.49, \, 1$). Vectors, scaled according to the reference arrow in each plot, depict the force fields $\textbf{f} = - \delta E/\delta \phi \varvec{\nabla } \phi$ needed to counterbalance the elastic reaction of the membrane in the given configuration, see our previous work for more details^22^. The D-symbols present in the $\alpha = 0.22, \, 0.34, \, 0.49, \, 1$ enlargements point out the configurations taken along the downhill stretches of the MEP of Fig. 1b, namely states where the elastic reaction $\textbf{f}_{e} = - \textbf{f}$ alone is sufficient to drive the fusion process. It is worth noticing that, as stated above, since the energy barrier is small and therefore the process can be thermally activated, actually there is no need of external force fields to drive the process even in the uphill stretches of the MEP, whose configurations reported in Fig. 2 are highlighted by the U-symbol. Overall, Fig. 2 shows that the forces are localized in the merging region and are more intense in the steepest stretches of the MEP. Furthermore, the change of direction of the vectors is a numerical confirmation that the saddle-point is placed between $\alpha = 0.33$ and $\alpha = 0.34$.

## Discussion

Bilayer elasticity predicts a very large fusion energy barrier of about $250 \, k_{B} T$, associated with a very steep energy path, and very intense forces localized in the merging region, all factors that are difficult to reconcile with the biological mechanisms involved in membrane fusion. In this work, we have shown that the sole local modification of the Gaussian modulus in the merging region drastically lowers the elastic energy barrier to $16 \, k_{B} T$ for a typical value of $k = 20 \, k_{B} T$, or to $39 \, k_{B} T$ for $k = 50 \, k_{B} T$. Although elasticity is commonly used to obtain valuable insights into membrane fusion^42,43^, it might be argued that these energies begin to be comparable to those that can emerge from the molecular detail that is inevitably missing in our approach. Despite this, our result is still relevant even if referring only to the elastic barrier. Indeed, in absence of a mechanism to lower it, the $250 \, k_{B} T$ elastic barrier would be the dominant one, and, for example, fusion proteins would be called upon to perform a formidable task in overcoming it. In fact, our results also show a MEP in accordance with experimental evidence, e.g. with an intermediate reminiscent of the hemifusion state^25^, which is also found in molecular dynamics simulations^44,45^. We also find that this intermediate is stabilized by the local modification of the Gaussian modulus, which therefore also substantially affect the pathway. In our previous work^22^, we showed that a uniform change of $k_{\textrm{G}}$ on the entire membrane was only able to rigid translate the fission branch, thus modifying the fusion energy barrier and the relative stability between the initial and final configurations, but not the shape of the pathway as in the present case.

A local modification of the Gaussian modulus might be relevant in several processes involving membrane fusion, as we shall now discuss. For example, calcium ions are capable of promoting fusion without the need for any proteins. Using molecular dynamics, Allolio and Harries^46^ showed that this ability is related to the generation of surface tension in the headgroup region, which in turn translates to a negative monolayer spontaneous curvature and an increased Gaussian modulus, see also Eq. (3). A locally varying $k_{\textrm{G}}$ may also play a role in the morphogenesis of neuroepithelial organoids, which has recently been shown^47^ to depend upon a uniform change in $k_{\textrm{G}}/k$. The local variation of the Gaussian modulus might also be related to the orientational ordering among oriented lipids^48–51^, which has been proposed to be relevant in the formation of thin necks that can subsequently break^52,53^. Another speculation can be made with regard to viral fusion. Indeed, viral proteins orchestrate at least the initial part of the fusion process by bringing the membranes in close proximity. The remainder of the process could proceed spontaneously due to thermal fluctuations, taking several minutes to complete^54^. Accordingly, the energy barrier for the fusion process should not exceed $40 \, k_{B} T$^37^, a fact which implies that viral proteins, in addition to having an apposition activity, should act as catalysts. From a physical point of view, this catalytic effect is compatible with a local modification of the Gaussian modulus. For example, influenza virus hemagglutinin (HA) protein is a $13.5 \, \text {nm}$ long trimer^55^, that can be viewed as a spring-loaded fusion machinery. When exposed to low pH, HA undergoes a series of conformational changes that lead to the insertion of its fusion peptides into the host membrane^56^. At that point, a refolding brings the two membranes in close contact and the fusion process can proceed towards the opening of a pore, passing through a hemifusion intermediate^57^. The insertion of a HA fusion peptide releases about $13 \, k_{B} T$, thus the action of three neighboring HA trimers is thought to generate enough energy to perturb the bilayer and overcome the dehydration barrier that keeps the two membranes apart^58^, a task also accomplished by increasing the protrusion of lipid tails^59^. Thus, this energy released upon insertion can be used to move from the zeroth stage of fusion^40^ to the following one. Once in close contact, fusion could proceed thermally due to the presence of HA. Hence, in addition to the mechanical work for the close apposition, peptides insertion ought to play a significant role for the additional catalytic effect that should lower the intrinsic fusion energy barrier, allowing fusion to be thermally activated. In this regard, it has been shown, both numerically^60^ and experimentally^61^, that the wild-type HA peptides promote negative Gaussian curvature, such as that of the catenoid-like necks. Therefore, an increase of $k_{\textrm{G}}$ in the merging region is compatible with the action of HA peptides and could catalyze the intrinsic fusion process due to the reduction of the intrinsic elastic barrier as shown in this work. Also the recently observed membrane thinning due to the aggregation of influenza peptides might contribute to modify the Gaussian modulus in viral fusion, Eq. (3). The same equation shows that if proteins modified the spontaneous curvature or the bending rigidity of the monolayers, the effect would still be to modify $k_{\textrm{G}}$. Beyond all these congruencies, the tendency of peptides to increase the Gaussian modulus also emerged in a recent molecular lipid model^62^, which also shows that the concomitant modification of *k* should be small. However, if the change in the elastic properties is localized in the merging region, the variation of the Gaussian modulus should be dominant. Indeed, in the merging region the leading forces are the Gaussian ones because of the high energy redistribution due to the Gauss-Bonnet theorem^22^. In any case, we found a MEP with a single hemifusion intermediate, like in HA mediated fusion^57,63,64^, and energy barriers that can be crossed in a time of several minutes, which is the time needed for viral infections to occur. Incidentally, viruses can have envelopes the size of our large vesicles^65^.

Of course, the main aim of this work is to show that a local change in $k_{\textrm{G}}$ can indeed have a strong impact on the intrinsic, elastic fusion path of two LUVs. We also discussed practical cases, both in presence and absence of proteins, where this modification might play a role. Clearly, there may also be instances where such a change is irrelevant. For example, one may think of membrane fusion induced by tension^66^. Nonetheless, even in this case $k_{\textrm{G}}$ could be affected^67^, albeit at the second order in $z_0$^14^.

## Methods

### Additional details on the free energy

As anticipated in the main text, $E_{\textrm{B}}[\phi ]$, Eq. (4), approaches the bending energy of the membrane in the sharp-interface limit^28,29^, while $E_{\textrm{G}}[\phi , \eta ]$, Eq. (6), approaches the Gaussian term^22^. Therefore, $E[\phi , \eta ]$ recovers the Canham–Helfrich free energy in the limit of small width-to-vesicle-extension ratio, $\lambda<< 1$. The complete derivation of this asymptotic behavior is developed in our previous work^22^ and is not affected by the dependence of $k_{\textrm{G}}$ on $\eta (\textbf{x})$. This auxiliary field is needed to discriminate between the small patch with varied Gaussian modulus and the remainder of the membrane. Following the work of Wang and Du^27^, $\eta (\textbf{x})$ identifies a field which is orthogonal to the phase-field $\phi (\textbf{x})$ representing the membrane. Furthermore, $\eta (\textbf{x}) \sim \tanh \left( a(\textbf{x})/\epsilon \sqrt{2} \right)$, where $a(\textbf{x})$ is the signed distance function from the $\eta = 0$ level set. Therefore, also $\eta (\textbf{x})$ assumes two limiting values $\pm 1$, in the regions inside and outside the $\eta = 0$ level set, respectively. Therefore, we say that the portion of the membrane lying where $\eta = -\, 1$ is the one with $k_{\textrm{G}} = k_{\textrm{G}_0} = - k$, while the small patch located within the $\eta = + \,1$ zone has the modified Gaussian modulus. Hence, we choose $k_{\textrm{G}}(\eta ) = k_{\textrm{G}_0} (1 - \eta )^2 / 4$ in Eq. (6). To make sure that the auxiliary field does indeed have a hyperbolic tangent profile, we add to the system the auxiliary energy

8 $$\begin{aligned} E_{\textrm{A}}[\eta ] = 10^{-3} k \, \dfrac{3}{4 \sqrt{2}} \, \epsilon \, \int _{\Omega } \Psi _{\textrm{A}}^2(\textbf{x}) \; dV, \end{aligned}$$

9 $$\begin{aligned} \Psi _{\textrm{A}} = \nabla ^2 \eta - \dfrac{1}{\epsilon ^2} \left( \eta ^2 - 1\right) \eta \;, \end{aligned}$$

that is a bending energy for $\eta$ with a very small bending rigidity, $10^{3}$ times smaller than that of the membrane. To ensure the orthogonality between the two fields, we use the following functional:

10 $$\begin{aligned} O[\phi , \eta ] = \int _{\Omega } | \varvec{\nabla } \phi \cdot \varvec{\nabla } \eta |^2 \; dV. \end{aligned}$$

As mentioned in the main text, all membrane configurations along the MEP have the same total surface area *A* and enclosed volume *V*. Indeed, since lipids are insoluble in water, the number of membrane lipids is conserved and a large tension is associated with the surface area change, implying that membrane bending cannot substantially modify *A*. At the same time, osmotic conditions constraint *V*^35^. In order to preserve these two quantities along the string, we used two functionals

11 $$\begin{aligned} A[\phi ] = \dfrac{3}{4 \sqrt{2}} \, \epsilon \, \int _{\Omega } \bigg [ \dfrac{\left( 1 - \phi ^2\right) ^2}{2\epsilon ^2} \, + \, |\varvec{\nabla } \phi |^2 \bigg ] \, dV, \end{aligned}$$

12 $$\begin{aligned} V[\phi ] = \int _{\Omega } \dfrac{(1 + \phi )}{2} \, dV, \end{aligned}$$

which recover the total surface area and enclosed volume, respectively, in the sharp interface limit, $\lambda<< 1$. Therefore, being $D_{\textrm{ve}} = \sqrt{A/\mathrm {\pi }}$, the reduced volume $v = V/(\mathrm {\pi } \, D_\text {ve}^3 / 6)$ is conserved along the MEP. Since the initial configuration ($\alpha = 0$) consists of two separate spheres with the same diameter $D = 146 \, \text {nm}$, $D_\text {ve}$ actually equals $\sqrt{2}D \approx 206 \, \text {nm}$ along the string and, therefore, $v = 1/\sqrt{2}$. Furthermore, we also want to preserve the surface area of the membrane patch with modified Gaussian modulus. For this purpose we use the functional:

13 $$\begin{aligned} P[\phi , \eta ] = \dfrac{3}{4 \sqrt{2}} \, \epsilon \, \int _{\Omega } \dfrac{(\eta + 1)^2}{4} \bigg [ \dfrac{\left( 1 - \phi ^2\right) ^2}{2\epsilon ^2} \, + \, |\varvec{\nabla } \phi |^2 \bigg ] \, dV. \end{aligned}$$

In conclusion, we have the modified energy

14 $$\begin{aligned} \begin{aligned} {\bar{E}}[\phi , \eta ] =&\, E[\phi , \eta ] \, + \, \gamma \left( A[\phi ]- A_0\right) + \dfrac{1}{2} M_1 \left( A[\phi ] - A_0\right) ^2 \, + \, \Delta p \left( V[\phi ] - V_0\right) + \dfrac{1}{2} M_2 \left( V[\phi ] - V_0\right) ^2 \\&+\, \gamma _p\left( P[\phi , \eta ]- P_0\right) + \dfrac{1}{2} M_3 \left( P[\phi , \eta ] - P_0\right) ^2 \, + \, E_{A}[\eta ] \, + \, M_4 (O(\phi , \eta ))^2 \;, \end{aligned} \end{aligned}$$

where $E[\phi , \eta ] = E_B[\phi ] + E_G[\phi , \eta ]$ is the membrane elastic energy which asymptotically behaves as the Canham-Helfrich one, Eq. (1), with a locally variable Gaussian modulus. Additional terms are needed when constraining to $A_0$, $V_0$, and $P_0$ the total surface area (Eq. (11)), enclosed volume (Eq. (12)), and surface area of the patch with modified Gaussian modulus, respectively. $M_1$, $M_2$, $M_3$ and $M_4$ are penalty constants, whereas $\gamma$, $\Delta p$ and $\gamma _p$ are updated at each time step following the *augmented Lagrangian method*^68^:

15 $$\begin{aligned}&\gamma ^{n+1} \; \; \, = \; \gamma ^n + M_1 \left( A\left[ \phi ^{n+1}\right] - A_{0}\right) , \end{aligned}$$

16 $$\begin{aligned}&\Delta p^{n+1} = \; \Delta p^n + M_2 \left( V\left[ \phi ^{n+1}\right] - V_{0}\right) , \end{aligned}$$

17 $$\begin{aligned}&\gamma _p^{n+1} \; \; \, = \; \gamma _p^n + M_3 \left( P\left[ \phi ^{n+1}\right] - P_{0}\right) . \end{aligned}$$

Therefore $\gamma$, $\Delta p$, and $\gamma _p$ are estimates of the Lagrange multipliers that improve at every time step. Orthogonality between fields is instead imposed directly through a pure penalty approach. Finally, it is worth saying that the energy associated with the auxiliary field does not affect in any substantial way the MEP of Fig. 1, indeed $\max _\alpha |\Delta {\bar{E}} (\alpha ) - \Delta E (\alpha )| < 0.086 \, k_{B} T$.

As we also discussed in our previous work^22^, the asymptotic Canham-Helfrich model is thought to hold^69^ for vesicles with $D_{\textrm{ve}} \ge 40 \; \ell _{\textrm{me}}$, being $\ell _{\textrm{me}}$ the lipid bilayer thickness; otherwise, higher-order terms in the energy density could make a significant contribution. For symmetric membranes, as those considered here, this limit safely reduces to $10 \; \ell _{\textrm{me}}$^69–71^. Therefore, the request is largely met by the LUVs considered in this work, since they have a characteristic length $D_{\textrm{ve}} \approx 206 \; \text {nm}$. Moreover, as also recently discussed by Duncan and Pezeshkian^72^, there is evidence that the Canham-Helfrich energy can work up to a length scale close to the thickness of the membrane^71^. In any case, we would like to stress that, as opposed to the Canham-Helfrich approach, our model explicitly takes into account a diffuse interface related to the membrane thickness. As also shown in this work, this interface plays a role in the highly curved merging region and reproduces experimentally observed configurations such as the hemifusion intermediate.

As a comment, we would like to stress that the discussed approach allows to simulate the full-scale evolution of topological transitions in LUVs, which is hardly achievable with molecular models. Moreover, this methodology can be exploited to explore the role of varying parameters like elastic constants and vesicle geometry to the extent that *gedankenexperimente* can be conceived. For example, electrostatic effects can be included, e.g., by following the prescription provided by Helfrich^73^. Hydrodynamics may be easily taken into account to investigate the transport of vesicles.

### Numerics

The simulation has been carried out using $N = 100$ images for the string^74^, and a $[0 \epsilon , \, 96 \epsilon ] \times [-\,245 \epsilon , \, 245 \epsilon ]$ computational domain in the $r-z$ plane (z-axial symmetry), with a grid of $144\times 735$ nodes per image, and $1/\lambda \approx 247.5$. We remind that the bilayer thickness $\ell _{\textrm{me}}$ is $6 \epsilon$ and $D_{\textrm{ve}} \approx 206 \; \text {nm}$. We used FFT-based spectral differentiation in a cell-centered grid, with a semi-implicit Euler single-step scheme to evolve the string, following the simplified string method algorithm^33^. Due to the presence of the auxiliary field, we actually evolved two fields to find the final MEP, namely

18 $$\begin{aligned} \dfrac{\partial \phi _i}{\partial t} = - M \dfrac{\delta {\bar{E}}}{\delta \phi _i} \;, \end{aligned}$$

and

19 $$\begin{aligned} \dfrac{\partial \eta _i}{\partial t} = - M \dfrac{\delta {\bar{E}}}{\delta \eta _i} \;, \end{aligned}$$

with $\; \; i = 1,... \,, N \,.$ As regards the reparametrization step^33^, the distance between the images and, thus, the total length of the pathway (used to calculate the normalized arc-length $\alpha$) are obtained with the metric induced by the norm of the vector valued function ($\phi _\alpha$, $\eta _\alpha$), namely $\Vert (\phi _\alpha , \eta _\alpha ) \Vert = \sqrt{\Vert \phi _\alpha \Vert _2^2 + \Vert \eta _\alpha \Vert _2^2}$, where the norm of the two fields are those induced by the standard $L_2$ inner product. Additional details on the numerical scheme and its convergence are also available in our previous work^22^. The functional derivative of the Gaussian energy, in presence of the locally variable $k_{\textrm{G}}(\eta (\textbf{x}))$ introduced in this work, reads:

20 $$ \begin{aligned}   \frac{{\delta E_{{\text{G}}} }}{{\delta \phi }} &  = \frac{{35}}{{8\sqrt 2 }}\frac{{\epsilon ^{3} }}{r}\left[ {12k_{{\text{G}}} \phi _{r} \left( {\phi _{{rz}}^{2}  - \phi _{{rr}} \phi _{{zz}} } \right) + \phi _{r} \phi _{z}^{2} k_{{{\text{G}}rr}}  + 6\phi _{r} (\phi _{r} \phi _{{rz}}  - \phi _{z} \phi _{{rr}} )k_{{{\text{G}}z}} } \right. \\     & \quad \left. { +\, {\mkern 1mu} \phi _{r}^{3} k_{{{\text{G}}zz}}  + 2\phi _{r}^{2} \phi _{z} k_{{{\text{G}}rz}}  + \left( {4\phi _{r} \phi _{z} \phi _{{rz}}  + \phi _{{rr}} \phi _{z}^{2}  - 5\phi _{r}^{2} \phi _{{zz}} } \right)k_{{{\text{G}}r}} } \right], \\  \end{aligned}  $$

where subscripts denote partial derivatives with respect to *r* and *z*. As a final remark, it is worth saying that even though results of Figs. 1 and 2 are reported assuming the typical value $-\, k_{\textrm{G}} = k = 20 \, k_{B}T$, they are actually independent of the choice of *k*^22^. In fact, provided that $-\, k_{\textrm{G}} = k$ in the unperturbed region, they can be rescaled with the value of *k*.

## Data Availability

The datasets generated during and/or analysed during the current study are available from the corresponding author on reasonable request.
